# SARS-CoV-2 Infection Impairs Oculomotor Functions: A Longitudinal Eye-tracking Study

**DOI:** 10.16910/jemr.17.1.2

**Published:** 2024-02-27

**Authors:** Xiaoting Duan, Zehao Huang, Shuai Zhang, Gancheng Zhu, Rong Wang, Zhiguo Wang

**Affiliations:** Zhejiang University, Hangzhou, China; Zhejiang Sci-Tech University, Hangzhou, China

**Keywords:** Eye movement, eye tracking, saccades, smooth pursuit, cognitive function, SARS-CoV-2 infection

## Abstract

Although Severe Acute Respiratory Syndrome Coronavirus 2 infection (SARS-CoV-2) is primarily recognized as a respiratory disease, mounting evidence suggests that it may lead to neurological and cognitive impairments. The current study used three eye-tracking tasks (free-viewing, fixation, and smooth pursuit) to assess the oculomotor functions of mild infected cases over six months with symptomatic SARS-CoV-2 infected volunteers. Fifty symptomatic SARS-CoV-2 infected, and 24 self-reported healthy controls completed the eye-tracking tasks in an initial assessment. Then, 45, and 40 symptomatic SARS-CoV-2 infected completed the tasks at 2- and 6-months post-infection, respectively. In the initial assessment, symptomatic SARS-CoV-2 infected exhibited impairments in diverse eye movement metrics. Over the six months following infection, the infected reported overall improvement in health condition, except for self-perceived mental health. The eye movement patterns in the free-viewing task shifted toward a more focal processing mode and there was no significant improvement in fixation stability among the infected. A linear discriminant analysis shows that eye movement metrics could differentiate the infected from healthy controls with an accuracy of approximately 62%, even 6 months post-infection. These findings suggest that symptomatic SARSCoV- 2 infection may result in persistent impairments in oculomotor functions, and the employment of eye-tracking technology can offer valuable insights into both the immediate and long-term effects of SARS-CoV-2 infections. Future studies should employ a more balanced research design and leverage advanced machine-learning methods to comprehensively investigate the impact of SARSCoV- 2 infection on oculomotor functions.

## Introduction

The COVID-19 pandemic has caused unprecedented disruptions to the global economy and inflicted over 770 million individuals worldwide (53). While SARS-CoV-2 is predominantly recognized as respiratory disease, mounting evidence also suggests nervous system disruption (2; 11; 28; 31; 33; 34), with notable clinical symptoms like sensorineural hearing loss (9), dysautonomia (12), anosmia (47), headache (35), etc. Autopsies on patients who died with COVID-19 also revealed that the SARS-CoV-2 RNA persisted throughout the brain, as late as 230 days following symptom onset (44). Similarly, recent studies have shown that SARS-CoV-2 infections may affect cognitive functions (4; 7; 15; 33; 56). For instance, mild SARS-CoV-2 infected cases show cognitive deficits that may relate to neuroinflammation (10).

Standardized neuropsychological tests are commonly used in clinical settings to assess cognitive functions (22). However, these tests require highly qualified individuals to administer and may fail to detect mild cognitive or neurological impairments that do not reveal themselves in observable symptoms or magnetic resonance imaging (MRI) scans, such as mild traumatic brain injuries (18). While researchers and medical practitioners continue to search for alternative diagnostic methods, there has been a recent surge in the utilization of eye-tracking technologies for detecting neurological impairments (24; 29; 45), assessing cognitive deficits ( 6; 37), and evaluating treatment responses ( 14; 32; 43; 49). Eye-tracking allows quick acquisition of objective and real-time analytics (5), and various eye-tracking tasks have been designed to capture eye movement metrics that help to reveal the neurological or psychological profile of an individual. For example, in a recent study, Zhang et al. (55) tested depressed patients with fixation stability, anti-saccade, and free-viewing tasks, and revealed noticeable differences in eye movement patterns between depressed participants and healthy controls. Hunfalvay et al. (17) also discovered that smooth pursuit tasks hold promise for detecting deficits in oculomotor functions stemming from concussions.

As noted, SARS-CoV-2 infection has been shown to burden the nervous system and impair cognitive functions (e.g., 56). However, in cases with mild symptoms, the impact of SARS-CoV-2 infection may be subtle and therefore may not be detected using traditional neuropsychological tests. The oculomotor system is closely linked to higher cognitive functions such as attention and executive control (48), and oculomotor function abnormalities have been studied as potential markers or proxies for cognitive impairments in various neurological and neuropsychiatric conditions. The current study examined whether impairments in oculomotor functions exist in recovered symptomatic SARS-CoV-2 infected participants immediately after recovery and at two- and six-month follow-ups. While oculomotor function tasks cannot yet replace cognitive assessments, the results of the present study should provide insights into the short- and long-term neural and cognitive impact of SARS-CoV-2.

## Methods

The research protocol reported in this paper was approved by a local ethics committee at the Center for Psychological Sciences, Zhejiang University. The experiments were carried out following the guidelines outlined in the Declaration of Helsinki, and all participants gave written informed consent before the assessments.

### Participants

The present study recruited 53 volunteers with self-reported prior SARS-CoV-2 infection and 25 volunteers self-reported having no prior SARS-CoV-2 infection from Zhejiang University. Those with prior SARS-CoV-2 infection all reported SARS-CoV-2 symptoms (see Table 1). For convenience, we will refer to these two groups of participants as SARS-CoV-2 infected and controls, respectively. Three SARS-CoV-2 infected participants were excluded from the analysis: two had been infected several months ago, one due to equipment failure. One participant in the control group was excluded because he reported being on medication for depressive disorder. So, in the initial assessment, there were 50 SARS-CoV-2 infected (27 females; *M* = 24.98 years, *SD* = 3.86) and 24 controls (20 females; *M* = 23.92 years, *SD* = 2.73). Statistical analysis showed that the ages of these two groups did not differ, *t*(72) = 1.56, *p* = 0.125, *Hedges' g* = 0.30. Among the SARS-CoV-2 infected, 45 (25 females/20 males, *M* = 25.07 years, *SD* = 3.89) returned to complete the 2-month follow-up assessment, and 40 (20 females/20 males; *M* = 24.90 years, *SD* = 4.07) returned to complete the 6-month follow-up assessment.

All participants reported having normal or corrected-to-normal visual acuity and having no other vision conditions (e.g., color blindness, amblyopia, high astigmatism, strabismus, cataracts, glaucoma, floaters, etc.). They were also required to report psychological conditions (e.g., depression, mania, schizophrenia, etc.) to the experimenter if there were any. No ophthalmologic evaluation was performed on the participants.

**Table 1.** Clinical symptoms experienced by the SARS-CoV-2 infected.

Clinical symptom	Percentage	Clinical symptom	Percentage
Fever	94%	Hyposmia or hypogeusia	42%
Headache	68%	Pneumonia	2%
Body aches	54%	Eye pain	2%
Asthenia	72%	Anorexia	2%
Dry throat	60%	Insomnia	2%
Rhinobyon	70%	Diarrhea	2%
Rhinorrhea	56%	Feel cold	2%
Cough	88%		

Note: Pneumonia included only those confirmed by computed tomography.

### Materials and Equipment

The participants completed three eye movement tasks in a quiet laboratory. The visual stimuli were presented against a gray background on a 21-inch ViewSonic LCD monitor. The refresh rate of this monitor was 144 Hz and its visible screen area measured 50° x 29.5° at a viewing distance of 57 cm (maintained with a chinrest). Stimulus presentation and eye-movement data recording were controlled by Python scripts running on a Windows 10 gaming laptop. Eye movement data were recorded at 1000 Hz with an EyeLink Portable Duo eye tracker (SR Research, Ottawa, Canada). The tracker was set to record binocular gaze data, but in rare occasions where only one eye could be reliably tracked, monocular gaze data was recorded. The typical tracking accuracy of this tracker was reported to be around 0.25°-0.5° (42).

### Procedure and Design

The initial assessment was administered shortly after the Chinese government lifted COVID-19 restrictions in late 2022. All participants completed a fixation task, a smooth pursuit task, and a free-viewing task. Details of the three eye movement tasks (i.e., fixation, smooth pursuit, and free viewing) are presented below.

*Fixation task*. Each participant completed four trials in the fixation task, two with and two without visual distractors. Visual distractors were presented to elicit intrusive saccades, which help assess the ability to maintain sustained attention (e.g., 5; 55). A drift-check was performed at the beginning of each trial. During the drift-check, a target was presented at the center of the screen and the participant was asked to press an assigned button when looking at the target to validate the accuracy of the calibration model (42). Following the drift-check, a fixation target (a white cross “+”) appeared at the center of the screen. In trials with distractors, a salient distractor (red disk; diameter = 0.44°) would appear at a random location 1.5° away from the fixation target. Participants were instructed to maintain their gaze on the fixation target and ignore the distractor. The visual distractor would show up on the screen at random intervals throughout a trial. Each trial lasted for 30 seconds, and the distractor would show up a total of 10 times. In trials without distractors, only the fixation target was presented at the screen center for 30 seconds.

*Smooth pursuit task*. Smooth pursuit tasks are commonly used for clinical assessment of neurological deficits (23). At the start of each trial, a drift-check target appeared 10° left or right of the screen center. Following a successful drift-check, a target stimulus (white disk; diameter = 0.69°) appeared at the same positions and began to move in a Lissajous pattern (frequency ratio = 3/4, amplitude = 10°). The participants were instructed to follow the target movement with their gaze. Each trial lasted for 48 seconds, and four trials were tested on each participant.

*Free-viewing task*. In the free-viewing task, the participants viewed 30 color pictures (31.38° x 18.11°) selected from publicly available datasets, HKU-IS (25), DUST (50), and PASCAL-S (26). The task consisted of thirty trials. Each trial started with a drift-check, followed by a picture that was presented for 5 seconds. The participant was instructed to freely view the picture.

The overall quality of life (QoL) of the participants was also assessed with the 36-item Short Form (SF-36; 30; 51). The same cohort of SARS-CoV-2 infected participants were re-assessed, using the same testing protocol, two and six months after the initial assessment.

### Data analysis

In the analysis, we first examined the immediate impact of SARS-CoV-2 infection by comparing the SARS-CoV-2 infected against the controls on various eye movement metrics. We then shifted focus to the SARS-CoV-2 infected and examined the evolving effect of SARS-CoV-2 infection on eye movement metrics over time. Lastly, linear discriminant analyses were performed to examine whether eye movement metrics can effectively differentiate individuals with a history of SARS-CoV-2 infection from controls.

Data analyses were performed in R 4.2.1 (38), and the effect size measure (*Hedges’ g*) reported in this paper was calculated with the *esc* package in R (27). Repeated measure ANOVAs, when needed, were performed using the *bruceR* package in R (3). Significant main effects observed in the ANOVA were further examined with Bonferroni-corrected post-hoc comparisons. The *t*-tests reported in this study were corrected using a method recommended by Ruxton (39). This approach involved an initial examination of the distribution of the dependent measure, followed by the performance of an unequal variance *t*-test on either ranked or unranked data, depending on whether the dependent measure exhibited a normal distribution or not. The *scikit-learn* library in python was applied to the linear discriminant analysis.

**Figure 1.**
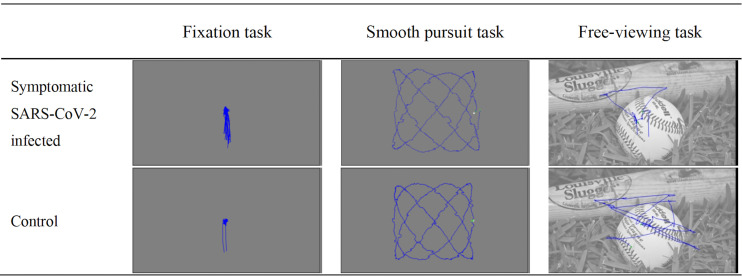
Sample eye movement recordings from the three oculomotor tasks

The present study recorded binocular gaze data, with a few exceptions where good binocular calibration could not be obtained (5, 5, and 8 instances in the smooth pursuit task, free-viewing task, and fixation task, respectively). Sample eye movement recordings from the three oculomotor tasks are presented in Figure 1. Various eye movement metrics that have been previously used in clinical studies were compared between the two groups (5). The eye movement metrics examined in all three tasks include fixation number, fixation duration (s), saccade number, saccade duration (ms), saccade amplitude (°), saccade average velocity (°/s), and saccade peak velocity (°/s). In the smooth pursuit task, we also examined velocity gain and root mean square error (RMSE), which reflect how closely the gaze followed the pursuit target. In the fixation task, we also examined two fixation stability metrics, i.e., bivariate contour ellipse area (BCEA) and RMSE. These eye movement metrics were extracted from the raw recordings using SR Data Viewer (version 4.3.1).

Saccades in the present study were identified with a velocity threshold of 30°/s and an acceleration threshold of 8000°/s^2^. It is important to note that blink saccades were excluded from the analysis. In the free viewing task, nearby fixations were merged based on an amplitude threshold of 1.0° and a duration threshold of 50 ms. Fixations flanking blinks were treated as separate ones, as the gaze position usually changes following blinks. The EyeLink event detection algorithm categorized smooth pursuit movements as “fixations”. In the smooth pursuit task, velocity gain was calculated individually for all fixations by dividing the average gaze angular velocity by the average target velocity and then aggregated for each trial.

## Results

### The immediate and evolving impact of SARS-CoV-2 infection

*Quality of life ratings (SF-36).* Both the SARS-CoV-2 infected and controls completed the SF-36 survey in the initial and follow-up assessments. The SF-36 domain scores of the three assessments are summarized in Table 2. In the initial assessment, the SARS-CoV-2 infected reported deterioration in several domains, including role limitation due to physical health, social functioning, bodily pain, role limitation due to emotional problems, and general health perceptions. Over the six months following the infection, the quality of life of the SARS-CoV-2 infected showed improvement across all SF-36 domains, except for self-perceived mental health, *F* = 0.08, *p* = 0.921.

*Eye movement metrics.* At the beginning of the recording session, participants were instructed to look at 9 fixation targets on the monitor sequentially to calibrate the tracker. No significant difference in the calibration result was observed between the controls (*M* = 0.37°, *SD* = 0.11) and the SARS-CoV-2 infected (*M* = 0.40°, *SD* = 0.17), *p* = 0.129. The eye movement metrics extracted from the tasks are summarized in Table 3.

**Table 2.** SF-36 domain scores of the SARS-CoV-2 infected and controls.

SF-36 domains	Mean (*SD*)					*P*-value				
	C_0_	T_0_	T_1_	T_2_		C_0_ vs. T_0_	C_0_ vs. T_1_	C_0_ vs. T_2_	[T_0_ T_1_ T_2_] **^#^**	
Physical functioning	94.17 (9.05)	89.50 (11.75)	93.07 (5.63)	94.38 (8.93)		0.050^+^ ↓	0.119	0.670	0.063^+^ ↑	
Role limitation due to physical health	91.67 (21.70)	19.50 (30.43)	80.68 (34.89)	71.25 (39.45)		< 0.001^***^ **↓**	0.125	0.018^*^ **↓**	<0.001^***^ **↑**	T_0_ < T_1_ = T_2_
Social functioning	87.08 (11.58)	75.75 (14.84)	81.19 (16.54)	81.44 (13.75)		< 0.001^***^ **↓**	0.206	0.150	0.041^*^ **↑**	T_0_ < T_2_
Bodily pain	84.83 (14.78)	58.88 (19.02)	76.66 (16.45)	79.45 (17.11)		< 0.001^***^ **↓**	0.070^+^ ↓	0.258	<0.001^***^ **↑**	T_0_ < T_1_ = T_2_
General mental health	68.83 (14.59)	65.28 (12.92)	65.82 (14.00)	66.40 (13.97)		0.227	0.535	0.415	0.921	
Role limitation due to emotional problems	72.22 (37.64)	40.00 (43.12)	68.18 (38.01)	75.83 (36.19)		0.002^**^ **↓**	0.655	0.716	<0.001^***^ **↑**	T_0_ < T_1_ = T_2_
Vitality	68.13 (12.05)	63.70 (14.14)	69.09 (12.35)	66.75 (14.44)		0.169	0.693	0.684	0.006^**^ **↑**	T_0_ < T_1_
General health perceptions	76.00 (11.47)	68.22 (16.10)	71.39 (14.79)	73.68 (17.45)		0.036^*^ **↓**	0.337	0.868	0.029^*^ **↑**	

Notes: C_0_ refers to the control group in the initial assessment, whereas T_0_ to T_2_ refers to the initial, 2-month, and 6-month assessments, respectively; ^**#**^ The ANOVAs are performed on the infected participants who completed *SF-36* in all three assessments, and the means and SDs are based on the participants who completed the questionnaire in each assessment. ^+^*p* < 0.1; ^*^p < 0.05; ^**^p < 0.01; ^***^p < 0.001.

In the initial assessment, a significant elevation in saccade amplitude, saccade average velocity, saccade peak velocity, and a fixation stability (RMSE), were observed among the SARS-CoV-2 infected in the fixation task, regardless of the presence of visual distractors. There was a decrease in fixation duration in the SARS-CoV-2 infected when no distractor was present. In the free-viewing task, the SARS-CoV-2 infected had fewer fixations than controls. The smooth pursuit task revealed no reliable difference between the two groups, all |*t*’s| ≤ 1.19, all *p*’s ≥ 0.243.

In the second-month follow-up (T_1_), the SARS-CoV-2 infected continued to exhibit slightly poorer fixation stability, as evidenced by marginally higher RMSE (with distractor) and log_10_(BCEA) (without distractor). Furthermore, there was a decrease in fixation duration (without distractor), coupled with an increase in saccade amplitude and average velocity (with and without distractor) among the SARS-CoV-2 infected in the fixation task. When compared to the control group, the SARS-CoV-2 infected also had a decrease in fixation number in the free-viewing task and an increase in velocity gain in the smooth pursuit task.

In the sixth-month follow-up (T_2_), there was a significant rise in saccade amplitude and average velocity, and a marginal increase in saccade peak velocity and RMSE among the SARS-CoV-2 infected in the fixation task (with distractor). There was a decrease in fixation and saccade number and an increase in fixation duration in the free-viewing task.

Over the six months following the infection ([T_0_ T_1_ T_2_] in Table 3), a significant increase in fixation duration, and decreases in fixation number, saccade amplitude, saccade average velocity, saccade peak velocity, and saccade number were observed among the SARS-CoV-2 infected in the free-viewing task. The smooth pursuit task, on the other hand, revealed an increase in velocity gain and a decrease in saccade peak velocity.

The saccade peak velocity to amplitude ratio (main sequence) was also examined. When comparing to the health controls, there was a decrease among the SARS-CoV-2 infected in the fixation task (with distractor) at T_0_ (*p* = 0.004), T_1_ (*p* = 0.033), and T_2_ (*p* = 0.029). Over the six months following infection, there was a change in peak velocity to amplitude ratio in the free-viewing task (*p* = 0.005). No other significant effect was observed in other tasks (all *ps* ≥ 0.176).

### Linear discriminant analysis

We also investigated whether the eye movement metrics could effectively differentiate between the SARS-CoV-2 infected and controls with linear discriminant analyses (LDA). The participants included in this analysis include the controls who completed all tasks in the initial assessment (*n* = 23; 20 females/3 males; *M* = 24.00 years, *SD* = 2.76), the SARS-CoV-2 infected who completed all tasks in the initial assessments (*n* = 49; 26 females/23 males; *M* = 25.12 years, *SD* = 3.77), and the SARS-CoV-2 infected that completed all tasks in the 2-month (*n* = 44; 24 females/20 male; *M* = 25.14 years, *SD* = 3.91) and 6-month (*n* = 40; 20 females/20 males; *M* = 24.90 years, *SD* = 4.07) follow-up assessments.

LDA was performed on the eye movement metrics exacted from individual experimental tasks and all experimental tasks combined. When considering data from all three experimental tasks, the linear discriminant analysis demonstrated the ability to differentiate between SARS-CoV-2 infected and controls with an accuracy of 59.72%, 59.70%, and 61.90% at the initial assessment (T_0_), 2-month (T_1_), and 6-month (T_2_) follow-up assessments, respectively. When examining individual tasks, the fixation task with distractors yielded discriminative accuracies of 65.28%, 53.73%, and 50.79% at T_0_, T_1_, and T_2_, respectively. In the fixation task without distractors, the discriminant accuracies reached 63.89%, 64.18%, and 60.32% at T_0_, T_1_, and T_2_, respectively. With data from the free-viewing task, the discriminative accuracies were 56.94%, 61.19%, and 58.73% at T_0_, T_1_, and T_2_, respectively. Meanwhile, with data from the smooth pursuit task, the discriminative accuracies were 58.33%, 53.73%, and 53.97%.

**Table 3.** Eye movement metrics extracted from three eye movement tasks.

Eye movement metrics	Mean (*SD*)					*p*-value				
	C_0_	T_0_	T_1_	T_2_		C_0_ vs. T_0_	C_0_ vs. T_1_	C_0_ vs. T_2_	[T_0_ T_1_ T_2_] **^#^**	
Fixation task (without distractor)										
Fixation number	25.37 (17.69)	32.05 (16.26)	30.48 (13.98)	27.87 (13.30)		0.081^+^ ↑	0.104	0.261	0.278	
Fixation duration (s)	2.30 (2.93)	1.21 (0.74)	1.30 (0.89)	1.47 (0.97)		0.037^*^ **↓**	0.066^+^ ↓	0.260	0.294	
Saccade duration (ms)	13.19 (4.78)	15.84 (6.91)	13.40 (4.28)	13.78 (5.59)		0.121	0.785	0.746	0.051^+^ ↓	
Saccade amplitude (°)	0.61 (0.65)	0.87 (1.27)	0.63 (0.41)	0.70 (0.89)		0.013^*^ **↑**	0.066^+^ ↑	0.143	0.320	
Saccade average velocity (°/s)	39.71 (12.84)	45.75 (18.47)	42.32 (10.01)	41.90 (16.86)		0.014^*^ **↑**	0.078^+^ ↑	0.360	0.377	
Saccade peak velocity (°/s)	53.18 (23.80)	64.54 (33.78)	56.27 (18.72)	56.92 (31.60)		0.014^*^ **↑**	0.182	0.347	0.263	
Saccade number	24.69 (17.88)	31.42 (16.18)	29.98 (14.02)	27.03 (13.39)		0.077^+^ ↑	0.114	0.275	0.234	
RMSE	0.56 (0.34)	0.83 (0.85)	0.65 (0.39)	0.61 (0.39)		0.042^*^ **↑**	0.120	0.179	0.146	
Log_10_(BCEA)	-0.69 (0.39)	-0.50 (0.50)	-0.57 (0.39)	-0.59 (0.35)		0.057^+^ ↑	0.093^+^ ↑	0.125	0.507	
Fixation task (with distractor)										
Fixation number	21.49 (15.33)	25.40 (12.55)	24.67 (13.41)	22.90 (13.73)		0.078^+^ ↑	0.168	0.549	0.949	
Fixation duration (s)	2.07 (1.33)	1.52 (0.85)	1.93 (2.28)	2.21 (2.35)		0.082^+^ ↓	0.137	0.632	0.353	
Saccade duration (ms)	13.63 (4.26)	14.43 (4.34)	14.89 (6.80)	14.30 (4.57)		0.457	0.662	0.554	1.000	
Saccade amplitude (°)	0.44 (0.17)	0.73 (0.55)	0.62 (0.43)	0.64 (0.43)		< 0.001^***^ **↑**	0.004^**^ **↑**	0.005^**^ **↑**	0.293	
Saccade average velocity (°/s)	37.51 (6.10)	43.83 (11.12)	42.70 (11.02)	42.56 (9.54)		0.007^**^ **↑**	0.022^*^ **↑**	0.015^*^ **↑**	0.741	
Saccade peak velocity (°/s)	49.83 (10.61)	60.94 (21.44)	58.15 (20.21)	58.23 (18.46)		0.019^*^ **↑**	0.084^+^ ↑	0.087^+^ ↑	0.673	
Saccade number	20.54 (15.30)	24.70 (12.53)	24.01 (13.61)	22.43 (14.11)		0.061^+^ ↑	0.132	0.467	0.983	
RMSE	0.44 (0.19)	0.58 (0.29)	0.59 (0.37)	0.54 (0.25)		0.031^*^ **↑**	0.075^+^ ↑	0.098^+^ ↑	0.399	
Log_10_(BCEA)	-0.74 (0.25)	-0.62 (0.33)	-0.63 (0.37)	-0.64 (0.32)		0.085^+^ ↑	0.263	0.176	0.770	
Free-viewing task										
Fixation number	17.63 (1.52)	16.89 (1.68)	16.58 (1.76)	16.59 (1.49)		0.067^+^ ↓	0.014^*^ **↓**	0.011^*^ **↓**	0.029^*^ **↓**	
Fixation duration (s)	0.25 (0.33)	0.26 (0.35)	0.27 (0.050)	0.27 (0.42)		0.365	0.120	0.032^*^ **↑**	0.003^**^ **↑**	T_0_ < T_2_
Saccade duration (ms)	38.86 (2.60)	38.90 (2.62)	39.09 (2.64)	38.71 (3.69)		0.850	0.602	0.539	0.921	
Saccade amplitude (°)	5.89 (0.81)	6.02 (0.83)	5.81 (0.66)	5.68 (0.65)		0.329	0.921	0.531	0.014^*^ **↓**	T_0_ > T_2_
Saccade average velocity (°/s)	136.42 (12.90)	139.91 (14.19)	134.57 (10.92)	133.59 (10.87)		0.303	0.562	0.381	0.002^**^ **↓**	T_0_ > T_1_ = T_2_
Saccade peak velocity (°/s)	230.20 (29.14)	240.17 (34.17)	226.42 (28.24)	225.63 (27.14)		0.205	0.613	0.542	<0.001^***^ **↓**	T_0_ > T_1_ = T_2_
Saccade number	14.68 (1.51)	14.51 (1.79)	14.02 (1.80)	13.91 (1.40)		0.665	0.116	0.052^+^ ↓	<0.001^***^ **↓**	T_0_ > T_2_
Smooth pursuit task										
Fixation number	71.20 (26.57)	70.85 (23.14)	73.01 (28.16)	70.86 (27.23)		0.957	0.795	0.961	0.928	
Fixation duration (s)	0.74 (0.29)	0.73 (0.28)	0.75 (0.36)	0.78 (0.37)		0.794	0.820	0.966	0.672	
Saccade duration (ms)	17.81 (5.04)	19.38 (6.75)	18.20 (8.85)	17.49 (5.80)		0.448	0.586	0.472	0.118	
Saccade amplitude (°)	0.99 (0.43)	1.31 (1.21)	1.04 (0.72)	1.13 (0.99)		0.267	0.858	0.935	0.101	
Saccade average velocity (°/s)	51.82 (8.99)	57.45 (18.61)	52.94 (13.18)	54.08 (16.14)		0.349	0.825	0.956	0.105	
Saccade peak velocity (°/s)	73.23 (17.99)	84.25 (33.46)	74.19 (24.82)	75.83 (29.23)		0.243	0.708	0.793	0.022^*^ **↓**	T_0_ > T_1_
Saccade number	70.22 (26.55)	70.08 (23.15)	72.24 (28.27)	69.98 (27.32)		0.982	0.773	0.973	0.931	
Velocity gain	1.00 (0.08)	1.03 (0.19)	1.10 (0.21)	1.06 (0.14)		0.934	0.009^**^ **↑**	0.245	0.004^**^ **↑**	T_0_ < T_1_
RMSE	1.00 (0.40)	1.12 (0.62)	1.08 (0.55)	1.12 (0.62)		0.475	0.748	0.385	0.847	

Notes: C_0_ refers to the control group in the initial assessment, whereas T_0_ to T_2_ refers to the initial, 2-month, and 6-month assessments of the SARS-CoV-2 infected, respectively. RMSE, root mean square error; BCEA: bivariate contour ellipse area. **^#^** The ANOVAs are performed on the infected participants who completed the tasks in all three assessments, and the means and SDs are based on the participants who completed the tasks in each assessment. ^+^*p* < 0.1; ^*^p < 0.05; ^**^p < 0.01; ^***^p < 0.001.

## Discussion

Previous studies have shown that the rich features derived from eye-tracking data can effectively distinguish clinical groups from healthy controls with exceptional accuracy (e.g., 5; 55). In the current study, we investigated both the immediate and long-term impact of SARS-CoV-2 infection on oculomotor functions with three eye-tracking tasks.

### The immediate impact of SARS-CoV-2 infection

During the initial assessment, the SF-36 survey, and three eye movement tasks were administered to the SARS-CoV-2 infected and controls. We observed significant group differences in multiple eye movement metrics in the free-viewing and fixation tasks.

In the free-viewing task, the SARS-CoV-2 infected exhibited a marginal decrease in the number of fixations. The number of fixations during free viewing has been associated with several mental disorders (schizophrenia, depression, etc.; e.g., 5; 16). Previous studies have also linked this metric with exploratory behaviors (e.g., 36). Few volunteers in the current study reported experiencing mental problems; the observed decline in fixation number among the SARS-CoV-2 infected is likely associated with impairments in exploratory eye movements.

In the fixation task, the SARS-CoV-2 infected exhibited an increase in saccade amplitude, saccade average velocity, saccade peak velocity, and RMSE, regardless of the presence of visual distractors. Furthermore, there was a marginal increase in the number of saccades. Saccades that occur during a prolonged fixation are also known as intrusive saccades and have been associated with attention-deficit / hyperactivity disorder (e.g., ADHD; 13). These observations suggest that the SARS-CoV-2 infected may suffer from impairments in attention and executive function to some extent.

Besides, the SARS-CoV-2 infected participants exhibited deteriorated health conditions in several SF-36 domains when compared to the healthy controls. These findings were not surprising, considering that the SARS-CoV-2 infected were surveyed soon after their recovery, and most of them had reported experiencing one or more physical symptoms associated with the infection (see Table 1 for details).

### Long-lasting oculomotor impairments due to SARS-CoV-2 infection

Following the initial assessment, most of the SARS-CoV-2 infected returned to the lab to complete the 2- and 6-month follow-up assessments. Over the six months following the SARS-CoV-2 infection, the SARS-CoV-2 infected showed overall improvements in health conditions across all SF-36 domains, except for general mental health. In the eye movement tasks, we observed an increase in fixation duration, coupled with a decrease in fixation number, saccade amplitude, saccade average/peak velocity, and saccade number among the SARS-CoV-2 infected in the free-viewing task. These observations suggest that the SARS-CoV-2 infected participants may have adopted a relatively focal processing mode (20), as previously observed in participants with mental disorders, potentially indicating cognitive dysfunctions (55). In the smooth pursuit task, there was an increase in velocity gain and a decrease in saccade peak velocity. The linear discriminant analysis further revealed that eye movement metrics extracted from all three tasks could differentiate between the SARS-CoV-2 infected and controls with accuracies of 59.72%, 59.70%, and 61.90% at the initial, 2-month, and 6-month assessments, respectively.

The above observations are consistent with recent studies revealing neurological and cognitive impairments following SARS-CoV-2 infection (e.g., 56), suggesting persistent alterations in oculomotor functions among the SARS-CoV-2 infected. The mechanisms that associate SARS-CoV-2 infection and cognition and brain health are unclear, but possible candidates may include cerebrovascular factors, dysregulated autoimmunity and neuroinflammation, direct viral invasion of the central nervous system, and psychological factors (56).

The reversibility of oculomotor impairments as a sequela of SARS-CoV-2 infection remains to be substantiated by further long-term research evidence.

### Limitations of the current study

It should be noted that previous researchers have found a series of ocular anomalies potentially arising from SARS-CoV-2 infection, including, retinal changes (19), ocular motor nerve palsy (21; 52), and visuo-constructive deficit (10). The current study did not assess these ocular anomalies due to a lack of resources. However, ocular anomalies due to SARS-CoV-2 infection are rare and last for a relatively short period (1); they are unlikely to confound the present findings.

The dysfunctions and complications resulting from SARS-CoV-2 infection could endure for at least six months among certain discharged patients (46). Several studies have also suggested that individuals may even develop new-onset long COVID-19 symptoms several months after their initial recovery (40). In the context of the present finding, previous research has highlighted the prevalence of cognitive dysfunctions and the prolonged recovery process among participants infected with or suspected of being infected with SARS-CoV-2 (8). We cannot rule out the possibility of reinfection with SARS-CoV-2 during the present study, as antibody levels tend to decrease over time, increasing the likelihood of reinfection (54). However, the current observation of persistent oculomotor dysfunctions is more likely attributable to long COVID-19, a phenomenon where individuals may experience persistent symptoms following the initial SARS-CoV-2 infection (41).

The relatively small and unbalanced sample may have contributed to the relatively low accuracy of the linear discriminant analyses. It is also possible that the eye movement metrics examined in the present study might not be sufficiently sensitive to discern the effects of SARS-CoV-2 infection. As such, caution is warranted when interpreting the results of the linear discriminant analysis. To address these limitations in future investigations, it is advisable to explore more advanced machine learning techniques, such as support vector machines.

Preliminary findings indicate that integrating a support vector machine (SVM) with principal component analysis (PCA) yields classification accuracies of 81.94%, 73.13%, and 74.19% at T_0_, T_1_, and T_2_, respectively.

One methodological limitation of this study is the lack of follow-up on the control group, as most of those in the control group were later infected during the surge of infections following the lift of COVID-19 restrictions. The present study also does not have a balanced design because there was a scarcity of uninfected volunteers who were willing to participate in the initial assessment.

### Conclusions

The current research found diverse deficits in eye movement metrics immediately following SARS-CoV-2 infection, persisting at 2 and 6 months later. A linear discriminant analysis shows that eye movement metrics could differentiate individuals with a history of SARS-CoV-2 infection from healthy controls with an accuracy of approximately 62%, even 6 months post-infection. The present findings suggest that symptomatic SARS-CoV-2 infection may result in impairments in oculomotor functions, and the employment of eye-tracking technology can offer valuable insights into both the immediate and long-term effects of SARS-CoV-2 infection.

### Ethics and declaration of conflict

All authors declare that the contents of the article are in agreement with the ethics described in http://biblio.unibe.ch/portale/elibrary/BOP/jemr/ethics.html and that there is no conflict of interest regarding the publication of this paper.

### Acknowledgments

We are grateful to the anonymous reviewers for their constructive comments on an early version of this study. Preliminary results of the initial assessment were presented as a poster at the 2023 Annual Meeting of the General and Experimental Psychology Division of the Chinese Psychological Society.

## References

[b1] Al-Sharif, E., Strianese, D., AlMadhi, N. H., D’Aponte, A., dell’Omo, R., Di Benedetto, R., & Costagliola, C. (2021). Ocular tropism of coronavirus (CoVs): A comparison of the interaction between the animal-to-human transmitted coronaviruses (SARS-CoV-1, SARS-CoV-2, MERS-CoV, CoV-229E, NL63, OC43, HKU1) and the eye. International Ophthalmology, 41(1), 349–362. 10.1007/s10792-020-01575-2 1573-2630 32880786 PMC7471531

[b2] Andalib, S., Biller, J., Di Napoli, M., Moghimi, N., McCullough, L. D., Rubinos, C. A., O’Hana Nobleza, C., Azarpazhooh, M. R., Catanese, L., Elicer, I., Jafari, M., Liberati, F., Camejo, C., Torbey, M., & Divani, A. A. (2021). Peripheral nervous system manifestations associated with COVID-19. Current Neurology and Neuroscience Reports, 21(3), 9. 10.1007/s11910-021-01102-5 1534-6293 33586020 PMC7882462

[b3] Bao, H.-W.-S. (2023). bruceR: Broadly Useful Convenient and Efficient R Functions (2023.9) [Computer software]. https://cran.r-project.org/web/packages/bruceR/index.html

[b4] Becker, J. H., Lin, J. J., Doernberg, M., Stone, K., Navis, A., Festa, J. R., & Wisnivesky, J. P. (2021). Assessment of cognitive function in patients after COVID-19 infection. JAMA Network Open, 4(10), e2130645. 10.1001/jamanetworkopen.2021.30645 2574-3805 34677597 PMC8536953

[b5] Benson, P. J., Beedie, S. A., Shephard, E., Giegling, I., Rujescu, D., & St Clair, D. (2012). Simple viewing tests can detect eye movement abnormalities that distinguish schizophrenia cases from controls with exceptional accuracy. Biological Psychiatry, 72(9), 716–724. 10.1016/j.biopsych.2012.04.019 0006-3223 22621999

[b6] Caldani, S., Gerard, C. L., Peyre, H., & Bucci, M. P. (2020). Pursuit eye movements in dyslexic children: Evidence for an immaturity of brain oculomotor structures? Journal of Eye Movement Research, 13(1). 10.16910/jemr.13.1.5 1995-8692 33828780 PMC7881873

[b7] Ceban, F., Ling, S., Lui, L. M. W., Lee, Y., Gill, H., Teopiz, K. M., Rodrigues, N. B., Subramaniapillai, M., Di Vincenzo, J. D., Cao, B., Lin, K., Mansur, R. B., Ho, R. C., Rosenblat, J. D., Miskowiak, K. W., Vinberg, M., Maletic, V., & McIntyre, R. S. (2022). Fatigue and cognitive impairment in Post-COVID-19 Syndrome: A systematic review and meta-analysis. Brain, Behavior, and Immunity, 101, 93–135. 10.1016/j.bbi.2021.12.020 1090-2139 34973396 PMC8715665

[b8] Davis, H. E., Assaf, G. S., McCorkell, L., Wei, H., Low, R. J., Re’em, Y., Redfield, S., Austin, J. P., & Akrami, A. (2021). Characterizing long COVID in an international cohort: 7 months of symptoms and their impact. EClinicalMedicine, 38, 101019. 10.1016/j.eclinm.2021.101019 2589-5370 34308300 PMC8280690

[b9] Degen, C., Lenarz, T., & Willenborg, K. (2020). Acute profound sensorineural hearing loss after COVID-19 pneumonia. Mayo Clinic Proceedings, 95(8), 1801–1803. 10.1016/j.mayocp.2020.05.034 1942-5546 32753155 PMC7275185

[b10] de Paula, J. J., Paiva, R. E. R. P., Souza-Silva, N. G., Rosa, D. V., Duran, F. L. S., Coimbra, R. S., Costa, D. S., Dutenhefner, P. R., Oliveira, H. S. D., Camargos, S. T., Vasconcelos, H. M. M., de Oliveira Carvalho, N., da Silva, J. B., Silveira, M. B., Malamut, C., Oliveira, D. M., Molinari, L. C., de Oliveira, D. B., Januário, J. N., . . . Romano-Silva, M. A. (2023). Selective visuoconstructional impairment following mild COVID-19 with inflammatory and neuroimaging correlation findings. Molecular Psychiatry, 28(2), 553–563. 10.1038/s41380-022-01632-5 1476-5578 35701598 PMC9196149

[b11] Desforges, M., Le Coupanec, A., Dubeau, P., Bourgouin, A., Lajoie, L., Dubé, M., & Talbot, P. J. (2019). Human coronaviruses and other respiratory viruses: Underestimated opportunistic pathogens of the central nervous system? Viruses, 12(1), 14. 10.3390/v12010014 1999-4915 31861926 PMC7020001

[b12] Eldokla, A. M., Mohamed-Hussein, A. A., Fouad, A. M., Abdelnaser, M. G., Ali, S. T., Makhlouf, N. A., Sayed, I. G., Makhlouf, H. A., Shah, J., & Aiash, H. (2022). Prevalence and patterns of symptoms of dysautonomia in patients with long-COVID syndrome: A cross-sectional study. Annals of Clinical and Translational Neurology, 9(6), 778–785. 10.1002/acn3.51557 2328-9503 35393771 PMC9110879

[b13] Falck-Ytter, T., Pettersson, E., Bölte, S., D’Onofrio, B., Lichtenstein, P., & Kennedy, D. P. (2020). Difficulties maintaining prolonged fixation and attention-deficit/hyperactivity symptoms share genetic influences in childhood. Psychiatry Research, 293, 113384. 10.1016/j.psychres.2020.113384 1872-7123 32823201

[b14] Fletcher-Watson, S., & Hampton, S. (2018). The potential of eye-tracking as a sensitive measure of behavioural change in response to intervention. Scientific Reports, 8(1), 14715. 10.1038/s41598-018-32444-9 2045-2322 30279422 PMC6168486

[b15] He, D., Yuan, M., Dang, W., Bai, L., Yang, R., Wang, J., Ma, Y., Liu, B., Liu, S., Zhang, S., Liao, X., & Zhang, W. (2023). Long term neuropsychiatric consequences in COVID-19 survivors: Cognitive impairment and inflammatory underpinnings fifteen months after discharge. Asian Journal of Psychiatry, 80, 103409. 10.1016/j.ajp.2022.103409 1876-2026 36549172 PMC9751007

[b16] Holmqvist, K., Nyström, M., Andersson, R., Dewhurst, R., Jarodzka, H., & van de Weijer, J. (2011). Eye tracking: A comprehensive guide to methods and measures (1st ed.). Oxford University Press.

[b17] Hunfalvay, M., Murray, N. P., Mani, R., & Carrick, F. R. (2021). Smooth pursuit eye movements as a biomarker for mild concussion within 7-days of injury. Brain Injury : [BI], 35(14), 1682–1689. 10.1080/02699052.2021.2012825 1362-301X 34894915

[b18] Hunt, A. W., Mah, K., Reed, N., Engel, L., & Keightley, M. (2016). Oculomotor-based vision assessment in mild traumatic brain injury: A systematic review. The Journal of Head Trauma Rehabilitation, 31(4), 252–261. 10.1097/HTR.0000000000000174 1550-509X 26291632

[b19] Invernizzi, A., Torre, A., Parrulli, S., Zicarelli, F., Schiuma, M., Colombo, V., Giacomelli, A., Cigada, M., Milazzo, L., Ridolfo, A., Faggion, I., Cordier, L., Oldani, M., Marini, S., Villa, P., Rizzardini, G., Galli, M., Antinori, S., Staurenghi, G., & Meroni, L. (2020). Retinal findings in patients with COVID-19: Results from the SERPICO-19 study. EClinicalMedicine, 27, 100550. 10.1016/j.eclinm.2020.100550 2589-5370 32984785 PMC7502280

[b20] Ito, J., Yamane, Y., Suzuki, M., Maldonado, P., Fujita, I., Tamura, H., & Grün, S. (2017). Switch from ambient to focal processing mode explains the dynamics of free viewing eye movements. Scientific Reports, 7(1), 1082. 10.1038/s41598-017-01076-w 2045-2322 28439075 PMC5430715

[b21] Jeong, G. U., Kwon, H.-J., Ng, W. H., Liu, X., Moon, H. W., Yoon, G. Y., Shin, H. J., Lee, I.-C., Ling, Z. L., Spiteri, A. G., King, N. J. C., Taylor, A., Chae, J. S., Kim, C., Ahn, D.-G., Kim, K.-D., Ryu, Y. B., Kim, S.-J., Mahalingam, S., & Kwon, Y.-C. (2022). Ocular tropism of SARS-CoV-2 in animal models with retinal inflammation via neuronal invasion following intranasal inoculation. Nature Communications, 13(1), 7675. 10.1038/s41467-022-35225-1 2041-1723 36509737 PMC9743116

[b22] Kanchan, A., Singh, A. R., Khan, N. A., Jahan, M., Raman, R., & Sathyanarayana Rao, T. S. (2018). Impact of neuropsychological rehabilitation on activities of daily living and community reintegration of patients with traumatic brain injury. Indian Journal of Psychiatry, 60(1), 38–48. 10.4103/psychiatry.IndianJPsychiatry_118_17 0019-5545 29736061 PMC5914261

[b23] Kullmann, A., Ashmore, R. C., Braverman, A., Mazur, C., Snapp, H., Williams, E., Szczupak, M., Murphy, S., Marshall, K., Crawford, J., Balaban, C. D., Hoffer, M., & Kiderman, A. (2021). Portable eye-tracking as a reliable assessment of oculomotor, cognitive and reaction time function: Normative data for 18-45 year old. PLoS One, 16(11), e0260351. 10.1371/journal.pone.0260351 1932-6203 34807938 PMC8608311

[b24] Levantini, V., Muratori, P., Inguaggiato, E., Masi, G., Milone, A., Valente, E., Tonacci, A., & Billeci, L. (2020). Eyes are the window to the mind: Eye-tracking technology as a novel approach to study clinical characteristics of ADHD. Psychiatry Research, 290, 113135. 10.1016/j.psychres.2020.113135 1872-7123 32505031

[b25] Li, G., & Yu, Y. (2015). Visual saliency based on multiscale deep features. 2015 IEEE Conference on Computer Vision and Pattern Recognition (CVPR), 5455–5463. 10.1109/CVPR.2015.7299184

[b26] Li, Y., Hou, X., Koch, C., Rehg, J. M., & Yuille, A. L. (2014). The secrets of salient object segmentation. 2014 IEEE Conference on Computer Vision and Pattern Recognition, 280–287. 10.1109/CVPR.2014.43

[b27] Lüdecke, D. (2019). esc: Effect Size Computation for Meta Analysis (0.5.1) [Computer software]. https://cran.r-project.org/web/packages/esc/index.html

[b28] Mao, L., Jin, H., Wang, M., Hu, Y., Chen, S., He, Q., Chang, J., Hong, C., Zhou, Y., Wang, D., Miao, X., Li, Y., & Hu, B. (2020). Neurologic manifestations of hospitalized patients with coronavirus disease 2019 in Wuhan, China. JAMA Neurology, 77(6), 683–690. 10.1001/jamaneurol.2020.1127 2168-6157 32275288 PMC7149362

[b29] McDonald, M. A., Holdsworth, S. J., & Danesh-Meyer, H. V. (2022). Eye movements in mild traumatic brain injury: Ocular biomarkers. Journal of Eye Movement Research, 15(2). 10.16910/jemr.15.2.4 1995-8692 36439911 PMC9682364

[b30] McHorney, C. A., Ware, J. E., Jr., & Raczek, A. E. (1993). The MOS 36-Item Short-Form Health Survey (SF-36): II. Psychometric and clinical tests of validity in measuring physical and mental health constructs. Medical Care, 31(3), 247–263. 10.1097/00005650-199303000-00006 0025-7079 8450681

[b31] Meinhardt, J., Radke, J., Dittmayer, C., Franz, J., Thomas, C., Mothes, R., Laue, M., Schneider, J., Brünink, S., Greuel, S., Lehmann, M., Hassan, O., Aschman, T., Schumann, E., Chua, R. L., Conrad, C., Eils, R., Stenzel, W., Windgassen, M., . . . Heppner, F. L. (2021). Olfactory transmucosal SARS-CoV-2 invasion as a port of central nervous system entry in individuals with COVID-19. Nature Neuroscience, 24(2), 168–175. 10.1038/s41593-020-00758-5 1546-1726 33257876

[b32] Mihara, M., Hayashi, A., Kakeue, K., & Tamura, R. (2023). Changes in saccadic eye movement and smooth pursuit gain in patients with acquired comitant esotropia after strabismus surgery. Journal of Eye Movement Research, 16(4). 10.16910/jemr.16.4.3 1995-8692 38567314 PMC10987044

[b33] Miners, S., Kehoe, P. G., & Love, S. (2020). Cognitive impact of COVID-19: Looking beyond the short term. Alzheimer’s Research & Therapy, 12(1), 170. 10.1186/s13195-020-00744-w 1758-9193 33380345 PMC7772800

[b34] Nagu, P., Parashar, A., Behl, T., & Mehta, V. (2021). CNS implications of COVID-19: A comprehensive review. Reviews in the Neurosciences, 32(2), 219–234. 10.1515/revneuro-2020-0070 2191-0200 33550782

[b35] Nazari, S., Azari Jafari, A., Mirmoeeni, S., Sadeghian, S., Heidari, M. E., Sadeghian, S., Assarzadegan, F., Puormand, S. M., Ebadi, H., Fathi, D., & Dalvand, S. (2021). Central nervous system manifestations in COVID-19 patients: A systematic review and meta-analysis. Brain and Behavior, 11(5), e02025. 10.1002/brb3.2025 2162-3279 33421351 PMC7994971

[b36] Ohya, T., Morita, K., Yamashita, Y., Egami, C., Ishii, Y., Nagamitsu, S., & Matsuishi, T. (2014). Impaired exploratory eye movements in children with Asperger’s syndrome. Brain & Development, 36(3), 241–247. 10.1016/j.braindev.2013.04.005 1872-7131 23668935

[b37] Panagiotidi, M., Paul, O., & Tom, S. (2017). Increased microsaccade rate in individuals with ADHD traits. Journal of Eye Movement Research, 10(1). 10.16910/10.1.6 1995-8692 33828642 PMC7141051

[b38] R Core Team. (2022). R: A language and environment for statistical computing. R Foundation for Statistical Computing, Vienna, Austria. URL https://www.R-project.org/

[b39] Ruxton, G. D. (2006). The unequal variance t-test is an underused alternative to Student’s t-test and the Mann–Whitney U test. Behavioral Ecology, 17(4), 688–690. 10.1093/beheco/ark016 1465-7279

[b40] Soriano, J. B., Murthy, S., Marshall, J. C., Relan, P., & Diaz, J. V.. (2022). A clinical case definition of post-COVID-19 condition by a Delphi consensus. The Lancet. Infectious Diseases, 22(4), e102–e107. 10.1016/S1473-3099(21)00703-9 1474-4457 34951953 PMC8691845

[b41] Spudich, S., & Nath, A. (2022). Nervous system consequences of COVID-19. Science, 375(6578), 267–269. 10.1126/science.abm2052 1095-9203 35050660

[b42] SR Research Ltd. (2022). EyeLink® Portable Duo User Manual (version 1.0.9). Oakville, Ontario: SR Research Ltd.

[b43] Stafford, T., Overton, P. G., & Hampsey, E. (2019). Can microsaccade rate predict drug response? Journal of Eye Movement Research, 12(6). 10.16910/jemr.12.6.12 1995-8692 PMC7962677 33828750

[b44] Stein, S. R., Ramelli, S. C., Grazioli, A., Chung, J.-Y., Singh, M., Yinda, C. K., Winkler, C. W., Sun, J., Dickey, J. M., Ylaya, K., Ko, S. H., Platt, A. P., Burbelo, P. D., Quezado, M., Pittaluga, S., Purcell, M., Munster, V. J., Belinky, F., Ramos-Benitez, M. J., . . . Chertow, D. S., & the NIH COVID-19 Autopsy Consortium. (2022). SARS-CoV-2 infection and persistence in the human body and brain at autopsy. Nature, 612(7941), 758–763. 10.1038/s41586-022-05542-y 1476-4687 36517603 PMC9749650

[b45] Tao, L., Wang, Q., Liu, D., Wang, J., Zhu, Z., & Feng, L. (2020). Eye tracking metrics to screen and assess cognitive impairment in patients with neurological disorders. Neurological Sciences, 41(7), 1697–1704. 10.1007/s10072-020-04310-y 1590-3478 32125540

[b46] The Lancet. (2020). Facing up to long COVID. Lancet, 396(10266), 1861. 10.1016/S0140-6736(20)32662-3 0140-6736 33308453 PMC7834723

[b47] Vaira, L. A., Salzano, G., Deiana, G., & De Riu, G. (2020). Anosmia and ageusia: Common findings in covid-19 patients. The Laryngoscope, 130(7), 1787–1787. 10.1002/lary.28692 1531-4995 32237238 PMC7228304

[b48] van Ede, F., Chekroud, S. R., & Nobre, A. C. (2019). Human gaze tracks attentional focusing in memorized visual space. Nature Human Behaviour, 3(5), 462–470. 10.1038/s41562-019-0549-y 2397-3374 31089296 PMC6546593

[b49] Vassallo, S., & Douglas, J. (2021). Visual scanpath training to emotional faces following severe traumatic brain injury: A single case design. Journal of Eye Movement Research, 14(4). 10.16910/jemr.14.4.6 1995-8692 34760060 PMC8575428

[b50] Wang, L., Lu, H., Wang, Y., Feng, M., Wang, D., Yin, B., & Ruan, X. (2017). Learning to detect salient objects with image-level supervision. 2017 IEEE Conference on Computer Vision and Pattern Recognition (CVPR), 3796–3805. 10.1109/CVPR.2017.404

[b51] Ware, J. E., Jr., & Sherbourne, C. D. (1992). The MOS 36-item short-form health survey (SF-36). I. Conceptual framework and item selection. Medical Care, 30(6), 473–483. 10.1097/00005650-199206000-00002 0025-7079 1593914

[b52] Wei, H., Yin, H., Huang, M., & Guo, Z. (2020). The 2019 novel cornoavirus pneumonia with onset of oculomotor nerve palsy: A case study. Journal of Neurology, 267(5), 1550–1553. 10.1007/s00415-020-09773-9 1432-1459 32100124 PMC7087661

[b53] World Health Organization. (n.d.). WHO Coronavirus (COVID-19) Dashboard. Retrieved October 12, 2023, from https://covid19.who.int

[b54] Ye, Y. (2023). China’s rolling COVID waves could hit every six months - infecting millions. Nature, 618(7965), 442–443. 10.1038/d41586-023-01872-7 1476-4687 37286677

[b55] Zhang, D., Liu, X., Xu, L., Li, Y., Xu, Y., Xia, M., Qian, Z., Tang, Y., Liu, Z., Chen, T., Liu, H., Zhang, T., & Wang, J. (2022). Effective differentiation between depressed patients and controls using discriminative eye movement features. Journal of Affective Disorders, 307, 237–243. 10.1016/j.jad.2022.03.077 1573-2517 35390355

[b56] Zhao, S., Toniolo, S., Hampshire, A., & Husain, M. (2023). Effects of COVID-19 on cognition and brain health. Trends in Cognitive Sciences, 27(11), 1053–1067. 10.1016/j.tics.2023.08.008 1879-307X 37657964 PMC10789620

